# Semi-automated approaches for interrogating spatial heterogeneity of tissue samples

**DOI:** 10.1038/s41598-024-55387-w

**Published:** 2024-02-29

**Authors:** Vytautas Navikas, Joanna Kowal, Daniel Rodriguez, François Rivest, Saska Brajkovic, Marco Cassano, Diego Dupouy

**Affiliations:** Lunaphore Technologies SA, Tolochenaz, Switzerland

**Keywords:** Proteomics, Computational biology and bioinformatics

## Abstract

Tissues are spatially orchestrated ecosystems composed of heterogeneous cell populations and non-cellular elements. Tissue components’ interactions shape the biological processes that govern homeostasis and disease, thus comprehensive insights into tissues’ composition are crucial for understanding their biology. Recently, advancements in the spatial biology field enabled the in-depth analyses of tissue architecture at single-cell resolution, while preserving the structural context. The increasing number of biomarkers analyzed, together with whole tissue imaging, generate datasets approaching several hundreds of gigabytes in size, which are rich sources of valuable knowledge but require investments in infrastructure and resources for extracting quantitative information. The analysis of multiplex whole-tissue images requires extensive training and experience in data analysis. Here, we showcase how a set of open-source tools can allow semi-automated image data extraction to study the spatial composition of tissues with a focus on tumor microenvironment (TME). With the use of Lunaphore COMET platform, we interrogated lung cancer specimens where we examined the expression of 20 biomarkers. Subsequently, the tissue composition was interrogated using an in-house optimized nuclei detection algorithm followed by a newly developed image artifact exclusion approach. Thereafter, the data was processed using several publicly available tools, highlighting the compatibility of COMET-derived data with currently available image analysis frameworks. In summary, we showcased an innovative semi-automated workflow that highlights the ease of adoption of multiplex imaging to explore TME composition at single-cell resolution using a simple slide in, data out approach. Our workflow is easily transferrable to various cohorts of specimens to provide a toolset for spatial cellular dissection of the tissue composition.

## Introduction

A plethora of biological events is coordinated by spatially orchestrated processes governing the dynamics of cell-intrinsic mechanisms and cell-to-cell interactions. The resolution of spatial patterning and cell organization becomes extremely relevant in heterogenous contexts such as the cancer ecosystem where the malignant lineages are only one of many players. The non-malignant counterparts that constitute the tumor microenvironment (TME), a complex sociological structure dominated by immune and stromal cells, along with vessels, and other mesenchymal components, highly contribute to cancer development and progression. Advances in spatial profiling technologies enable the precise characterization of molecular and cellular details^1,2^. Thus, new opportunities to reach unprecedented insights about phenotypic interactions and to open new avenues for the study of physio-pathological events are emerging. The deployment of such technologies usually poses significant challenges. The adoption barriers start with assay optimization, design, and image analysis. They also include the handling and processing of complex and heavy in size computational datasets. On top of that, amplifying the number of spatial features extracted from a minimal number of biological samples entails additional hurdles. While a fraction of these roadblocks emerges across different methodologies such as the preservation and the structural heterogeneity of tissue specimens, other confounding variables heavily depend upon the technology toolbox adopted for the spatial analysis. Choosing the image analysis toolset can also require strenuous efforts, especially finding the right balance between solution cost and complexity. On top of that, the adaptation and integration of open-source software solutions often require basic coding skills, minimal IT infrastructure, and adequate expertise that is required to identify and exploit the most appropriate methodology for advanced tissue phenotyping at single-cell level. The need for standardization in the multiplex immunofluorescence field is well-recognized with efforts being undertaken to standardize the guidelines for assays and image analysis process^3,4^. Recently, image analysis pipelines were suggested for spatial biology assays such as imaging mass cytometry^5^ and spatial transcriptomics^6^. For instance, an open-source computational pipeline, MCMICRO, was proposed as a standardized workflow^7^, which guides the user through all analysis steps to extract single-cell data from whole-slide images acquired using different spatial omics modalities. Similarly, efforts are being undertaken to render image analysis more technology-agnostic while focusing on the spatial context of the data, such as the discovery of cell niches^8^. Independently, the sequential immunofluorescence (seqIF) protocol that generated data for this study^9^ has been used to characterize inflamed mucosa^10^, pancreatic^11^, prostate^12^ and brain tumors^13–15^, but the dedicated set of open-access tools that support single-cell analysis has not been suggested so far.

Here, we describe an end-to-end workflow, composed of established open-source libraries, as the analysis tools for multi-layered spatial proteomic profiles generated on a wide range of biological tissues and conditions by seqIF using the Lunaphore COMET platform^9^. We further introduce the potential of our approach for any biological dataset generated in the OME-TIFF format, and its versatility to address key questions related to spatial biology including, but not limited to, cell phenotyping, distance proximity, and the detection of enriched cell-to-cell adjacencies. Our proposed solution integrates a new data-driven approach to clean the dataset from false positives thereby reducing the erroneous depictions for the rare cell entities. Our semi-automated supervised approach based on marker intensity serves to quantify cell phenotypes of interest rapidly and efficiently using both supervised and unsupervised phenotyping approaches.

## Methods

### Next-generation tissue microarray construction (ngTMA)

ngTMA was developed at the Institute of Tissue Medicine and Pathology, University of Bern, using tissues obtained from patients who provided informed consent for research purposes, in compliance with the Federal Act on Research Involving Human Beings dated 30 September 2011, commonly referred to as the Human Research Act (HRA). Specifically, this was done in accordance with Article 16 and Article 17 of the HRA, which mandates obtaining informed written consent from patients participating in research projects, permitting the use of their biological material for general research purposes. Permission to utilize the tissue material was granted by the Ethics Committee of the Canton of Bern, and the collection of these tissues was conducted in strict accordance with the HRA. The details of the construction of tissue microarrays (TMAs) have been described previously^16,17^. The cores used in the TMA were 0.6 mm in diameter. The TMA sample used in the study was composed of matched primary and metastatic tumor cores of lung cancer (Fig. 2A).

### Hyperplex staining and whole-slide imaging

Formalin-fixed paraffin-embedded (FFPE) slide was preprocessed with PT Module (Epredia) with Dewax and HIER Buffer H (TA999-DHBH, Epredia) for 60 min at 102 °C. Subsequently, the slide was rinsed and stored in a Multistaining Buffer (BU06, Lunaphore) till use. The 20-plex protocol template was generated using the COMET Control Software, and reagents were loaded onto the device to perform the fully automated sequential immunofluorescence (seqIF) protocol^9^. The nuclear signal was detected with DAPI (Thermo Scientific, cat no: 62248, 1/1000 dilution) by dynamic incubation of 2 min or by complementing secondary antibody cocktails with DAPI. For all staining cycles, the dynamic incubation time of primary antibody mixes was set to 4 min, while the dynamic incubation time of secondary antibodies and DAPI cocktails was set to 2 min. All primary antibody cocktails were diluted in Multistaining Buffer (BU06, Lunaphore), except for the CD31-aSMA mix that was diluted in 1% AURION BSA-c (Aurion). For each imaging cycle, the following exposure times were used: DAPI 80 ms, TRITC 400 ms, Cy5 200 ms. The elution step duration was set to 2 min for each cycle and was performed with Elution Buffer (BU07-L, Lunaphore). The quenching step was set to 30 s and was performed with Quenching Buffer (BU08-L, Lunaphore). The imaging step was performed with Imaging Buffer (BU09, Lunaphore). Primary antibody details can be found in Table 1. Alexa Fluor Plus 647 goat anti-mouse (Thermo Scientific, cat no: A32728, 1/200 dilution) and Alexa Fluor Plus 555 goat anti-rabbit (Thermo Scientific, cat no: A32732, 1/100 dilution) or Alexa Fluor Plus 647 goat anti-rabbit (Thermo Scientific, cat no: A32733, 1/200 dilution) and Alexa Fluor Plus 555 goat anti-mouse (Thermo Scientific, cat no: A32727, 1/100 dilution) secondary antibody mixes were used. Once the experiment was completed, a raw OME-TIFF file was generated by the COMET Control software for downstream analysis.

**Table 1:** Details on biomarker panel, image acquisition and data used for analysis.

Cycle	Marker	Fluorescence channel	Antibody clone, supplier	Cellular compartment	Max intensity value displayed in Fig. 2C
1	FoxP3	Cy5	SP97, Thermo Scientific	Nucleus	40,000
	CD68	TRITC	KP1, Thermo Scientific	Cytoplasm	50,000
2	aSMA	Cy5	1A4, Lunaphore	Cytoplasm	50,000
	CD31	TRITC	EP3095, Abcam	Cytoplasm	40,000
3	CD38	Cy5	SP149, Cell Marque	Cytoplasm	65,535
	IDO-1	TRITC	V1N3IDO, Thermo Scientific	Nucleus	40,000
4	S100	Cy5	4C4.9, Thermo Scientific	Cytoplasm	40,000
	CD11c	TRITC	EP1347Y, Abcam	Cytoplasm	40,000
5	PD-L1	Cy5	IHC411, GenomeMe	Cytoplasm	40,000
	ki67	TRITC	MIB-1, Dako	Nucleus	30,000
6	CD8	Cy5	4B11, Biorad	Nucleus	30,000
	PD-1	TRITC	EPR4877(2), Lunaphore	Cytoplasm	20,000
7	CD4	Cy5	EPR6855, Abcam	Nucleus	25,000
	PanCK	TRITC	AE1/AE3, Dako	Cytoplasm	40,000
8	CD3	Cy5	MRQ-39, Cell Marque	Nucleus	40,000
	CD20	TRITC	L26, Lunaphore	Cytoplasm	40,000
9	CD16	Cy5	SP175, Cell Marque	Cytoplasm	40,000
	HLA-DR	TRITC	TAL-1B5, Santa Cruz	Cytoplasm	50,000
10	Vimentin	Cy5	SP20, Abcam	Cytoplasm	40,000
	CD45	TRITC	PD7/26+2B11, Dako	Cytoplasm	30,000

### Image pre-processing

The final step of the COMET protocol consists of alignment, stitching, flat-field correction, and generation of output 16-bit OME-TIFF images. They are executed in the COMET Control software after the automated seqIF protocol execution and data acquisition. Pixel-wise autofluorescence correction was performed using Horizon Viewer software for each marker separately. Autofluorescence images acquired before each imaging cycle were used for the correction to minimize the occurrence of background subtraction artifacts. This also allowed us to ensure the minimal deviation of the fluorescence intensity values that might be caused by the photobleaching of autofluorescent tissue structures over 21 imaging cycles. The pre-processed stack was then exported and is available as Supplementary Data: https://lunaphore.com/download-center-tma-downstream-analysis/.

### Image segmentation

The DAPI image was used to segment cell nuclei, using a pre-trained StarDist nuclei segmentation model^18,19^. The model was pre-trained in-house using 12,138 manually annotated nuclei from various tissues imaged with the COMET platform. The custom-trained StarDist model allowed us to achieve approximately 15% higher segmentation precision. The estimated precision and F1 scores, computed using the validation dataset previously unseen by the model, were 0.92 and 0.83, respectively. Segmentation was performed in QuPath^20^ software. The segmented nuclei were dilated by 5 pixels (1.15 µm) to approximate the cell boundaries. The corresponding mean expression values were calculated from the segmentation masks for each of the fluorescence channels. The full expression table composed of 68,801 detected cells was then exported as a .csv file and used for further analysis. Nuclei segmentation masks were used to calculate the mean pixel values for FoxP3, IDO-1, ki67, CD3, CD4 and CD8 markers, for all other markers, the mean pixel values in the approximated cytoplasm compartments were used (Table 1). The proportion of PanCK-positive tissue was estimated by dividing the area of the whole TMA tissue by the area defined by a global threshold on the PanCK image.

### Data filtering

Before performing cell type assignment, the single-cell detections were filtered as described in Supplementary Figs. 1 and 2. In short, the erythrocytes were first excluded by performing unsupervised clustering of the expression table from a non-background subtracted image stack (Supplementary Fig. 1). Mean values of nuclei masks from all the markers, including the two first autofluorescence channels were used. For erythrocyte detection, each column of the data was normalized by subtracting the median and dividing by the standard deviation. Dimensionality reduction was then performed using uniform manifold approximation and projection (UMAP) algorithm implemented in the Scanpy framework^21^. For UMAP n_neighbors parameter was set to 40 with a min_dist parameter set to 0.5. The Leiden clustering was performed using a resolution parameter set to 1. Clusters with high expression levels in all channels (Supplementary Fig. 1B) were considered as clusters mainly composed of erythrocytes as it is visualized in Supplementary Fig. 1C,D. Visual examination confirmed accurate erythrocyte detection and 3432 detections were excluded for further processing. For further data cleaning, detections were filtered based on the 4 parameters that were measured for each annotation: StarDist detection probability, DAPI mean intensity, detection area and circularity as described in Supplementary Fig. 2. In short, the distributions of the corresponding measurements were then filtered excluding 5% lowest values. For area, cells with the 0.1% highest area were also excluded. 10,797 cells were removed from the dataset with the described filtering approach. The impact of the filtering procedure on the subsequent unsupervised data clustering was further examined in Supplementary Fig. 6. A total of 55,063 cells were used for further analysis.

### Supervised phenotyping

To perform a supervised cell type assignment, a binary tree classifier based on the expression of 14 markers was used. For performing a rule-based classification, the expression table was binarized by a custom intensity-based thresholding approach: each column of the pre-filtered expression table was winsorized with the upper limit of 0.01% to remove the outliers and then the data was Z-normalized by subtracting the mean and dividing by the standard deviation. To remove the user bias, automatic thresholding was performed based on the detected background parameters. The background was set to the lowest intensity peak in the Z-normalized data and the positivity threshold was set as 6σ from the position of the peak. The σ was defined as FWHM/2.355 and calculated for each marker separately. Finally, the decision tree-based classification was based on the pre-defined ruleset which is summarized in Fig. 3A. Data was visualized using Squidpy and Matplotlib frameworks^21,22^. Cell-type annotations were also examined in QuPath as image overlays.

For processing with the Astir framework, the expression table was filtered as described in Data Filtering and normalized using the arcsinh transformation with a factor of 150. The marker table based on the same rules as described in Fig. 3A was used as an input for the automated cell type assignment algorithm. After cell-type fitting, cells with an assignment probability < 0.5 were defined as Unknown.

### Unsupervised phenotyping

The expression table for unsupervised phenotyping was normalized as for supervised classification. The UMAP was computed with the n_neighbors parameter set to 40, and the min_dist parameter set to 0.5. The Leiden clustering was performed using a resolution parameter set to 0.8. A total number of 20 clusters were detected with the procedure described. The neighboring clusters with analogous PanCK expression pattern were merged into a metacluster (tumor cells) as described in Supplementary Fig. 8 to better reflect the data. The cluster with false-positive PD-1 cells was also removed based on the morphological features of the cells as described in Supplementary Fig. 9. Finally, the cell types were determined based on the Z-normalized expression table and data was visualized using Squidpy and Matplotlib frameworks^21,22^. Cell-type annotations were also examined in QuPath as image overlays.

### Spatial analysis

Cells that were classified in an unsupervised manner were further examined spatially, by using the spatial analysis features of a Squidpy framework^21^. The expression table was filtered based on the pre-determined cancer type: metastatic or primary for a comparison further described in Fig. 5. The graph from spatial coordinates was constructed using spatial_neighbors method with coord_type parameter set to generic. The neighborhood enrichment score (Fig. 5B) and interaction (Fig. 5D) matrices were then computed separately for both types of tissues. Finally, the co-occurrence scores for cells of interest (Fig. 5C) were calculated for each of the cores separately and averaged^21^.

### Ethical approval

This study has been approved by the Commission cantonale (VD) d'éthique de la recherche sur l’être humain (CER-VD), project ID: 2022-01489.

## Results

### Sequential immunofluorescence (seqIF) approach for spatial proteomics

**Figure 1:**
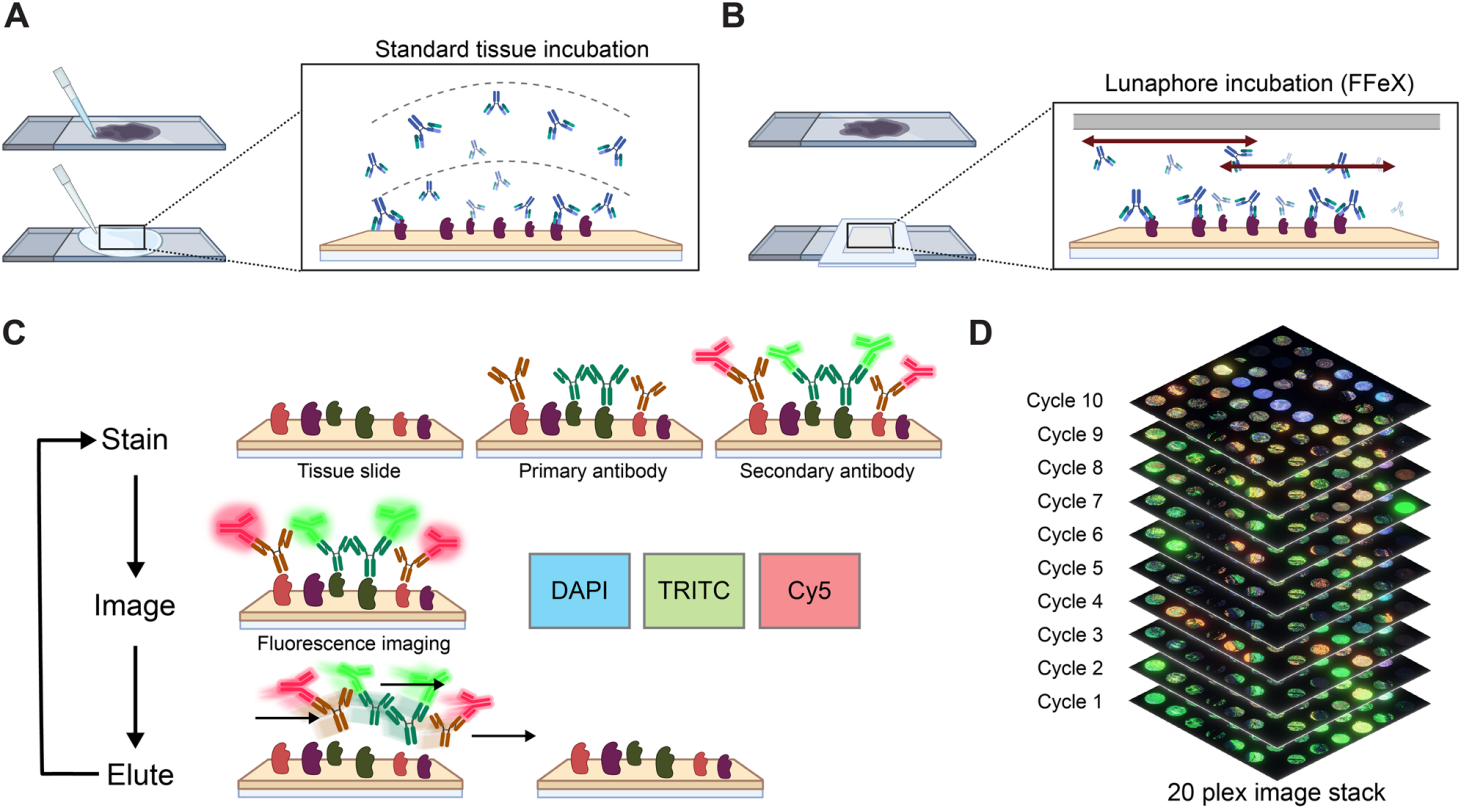
COMET platform automated workflow ensures “slide in, data out” approach. (**A**,**B**) In contrast to the passive diffusion of reagents happening during static incubation (**A**), the Fast-fluidic exchange (FFeX) technology achieved by the imaging microfluidic chip ensures active distribution of reagents and the possibility of a dynamic incubation over the surface of tissue sections (**B**). The homogeneous distribution of reagents ensures high-quality staining that is achieved in the scale of seconds^9^. Schematics were created with BioRender.com. (**C**) The COMET platform automates the sequential immunofluorescence (seqIF) protocol, where tissue samples can undergo 20 cycles of staining-imaging-elution. A pre-processed tissue (i.e.: tissue section that underwent deparaffinization and heat-induced epitope retrieval process offline) is sequentially stained with primary and secondary antibodies (2 antigens at a single step) together with a DAPI counterstaining and then imaged using a three-color fluorescence microscope. After imaging, an elution procedure is performed to remove the antibody complexes, and the process is repeated for another cycle. Schematics were created with BioRender.com. (**D**) 20-plex fluorescence image stack which was used for analysis is shown. COMET hyperplex images are stored as co-registered multi-layer image files using a generic OME-TIFF format.

To visualize the composition of tumoral tissues, we used the seqIF protocol on COMET (Fig. 1). The microfluidic setup was previously described and characterized^23^, with an adaptation of the scanning area to 81 mm^2^. COMET platform protocols are based on the fast fluidic exchange (FFeX) technology^24^ (Fig. 1B) that yields in ultra-fast and efficient antibody-based staining (approximately 15 min per single staining step of 2 markers), followed by imaging (approx. 30 min per marker) and elution (approx. 10 min per marker) (Fig. 1C). The immunostaining reaction, that occurs within the closed chamber formed in between the imaging window of the microfluidic chip and the histological slide, is precisely controlled via the automated system and the final signal reliably reflects the amount of antigen present in the tissue^25^. The seqIF protocol resulted in a co-registered multi-layer OME-TIFF image containing the following layers of information: nuclei signal acquired in the DAPI channel, intrinsic tissue autofluorescence images acquired in both TRITC and Cy5 channels, 20 single biomarker images. Additionally, we acquired the autofluorescence images after each elution cycle that allowed us to precisely monitor the evolution of autofluorescent signal and elution efficiency over the different cycles of the protocol^9^, and to perform accurate background subtraction (see methods chapter: Image pre-processing). COMET images from every cycle were stitched and automatically aligned within the COMET Control software, and the output file was ready for qualitative assessment and quantitative analysis at the end of the seqIF protocol (Fig. 1D).

**Figure 2:**
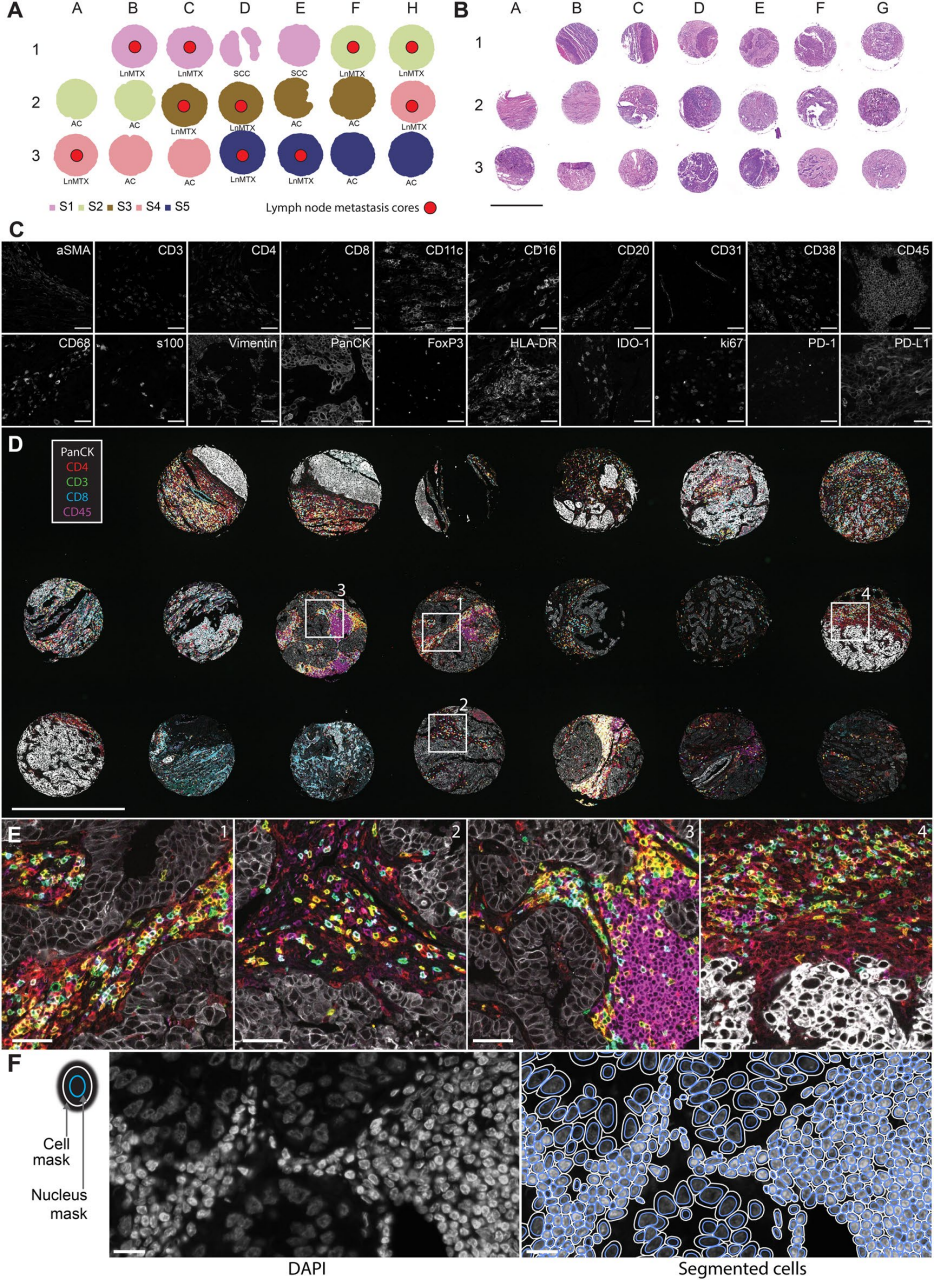
COMET enables high-quality and high-throughput data acquisition for multiple markers which can be used to segment cells and to extract single-cell data accurately for subsequent phenotype analysis. (**A**) A map of a ngTMA used for the hyperplex staining. The panel consists of cores from primary lung squamous cell carcinoma (SCC), primary lung adenocarcinoma (AC) and lung cancer lymph node metastasis (LNmtx) tissue samples from 5 different cases (specimens S1–S5). (**B**) Whole slide image (WSI) of the same TMA section stained with hematoxylin and eosin. Scale bar: 1 mm. (**C**) Background subtracted single channel zoom-in fluorescence images of all markers in the panel (see Table 1 for panel composition and staining conditions). Images are displayed using the same displayed minimal intensity value (0) and corresponding maximum intensity values (see Table 1). Scale bars: 50 µm. (**D**) An overlayed multicolor visualization of the fluorescence image of 5 markers from the panel (white: PanCK, red: CD4, green: CD3, blue: CD8, magenta: CD45). Scale bar: 1 mm. (**E**) Zoom-in into images from panel D, depicting (1–2) stroma-tumor interface, (3) accumulation of immune cells in the tumor stroma (3) and (4) accumulation of macrophages near the tumor stroma. Scale bar: 50 µm. (**F**) DAPI image with corresponding nuclei annotations, showing single-cell nuclei segmentation with a pre-trained StarDist^18^ model. The segmented nuclei were further dilatated by 5 pixels to approximate the cellular membrane. All scale bars: 20 µm.

To challenge the quality of data produced by the COMET device, we used a sample dataset that encompasses several specimens with a heterogenous tissue composition. To efficiently address this challenge, we used ngTMA containing both primary lung tumor samples and the corresponding lymph node metastasis specimens (Fig. 2A,B). The 20-plex panel consisted of biomarkers directly targeting immune cells (CD3, CD4, CD8, CD11c, CD16, CD20, CD31, CD45, CD68, FoxP3, HLA-DR), non-immune tumor microenvironment (aSMA, CD31, PanCK, S100, Vimentin), features of the immunosuppressed microenvironment (IDO-1, PD-1, PD-L1) and the proliferation marker ki67 (Fig. 2C). The specificity of detection of a single biomarker was evaluated according to internal standard guidelines^9^. For all biomarkers, the protocol resulted in high-quality staining that allowed to detect both signal positive and signal negative areas (Fig. 2C–E). Additional images were also acquired after each elution step allowing to assess the elution efficiency that was qualitatively deemed as excellent for all the markers based on quality-control criteria previously reported^9^. Once the initial quality control of the final image was passed, we moved toward the downstream image data analysis.

### Nuclei-based cell segmentation and data filtering

We based our analysis on a single-cell feature extraction, which in turn required a reliable cell segmentation approach (Fig. 2F). In the first step of the workflow, single-cell detection was performed based on the DAPI staining. We applied the StarDist method^18,19^ to delineate the single-cell annotations. To improve the segmentation results, we internally generated a dataset of 12,138 manually annotated nuclei from a heterogeneous dataset of 234 image crops extracted from COMET images of several tissue types. The validation dataset consisted of 1192 nuclei and was carefully crosschecked internally by manual curation. Training of the model with this dataset was harmonized as per the guidelines provided by the authors^18^. The model trained in-house showed a clear trend toward better performance when compared with the standard StarDist model (see methods section: Image segmentation). Once trained, the model was used to generate single-cell masks for the next steps of analysis. Subsequently, the annotation of nuclei was expanded by 5 pixels for each of the cells to obtain a proper cell delineation. The measurements of fluorescent signal intensities and the corresponding detected features were exported from QuPath^20^ for 68,801 segmented cells stemming from a single raw TMA image.

To ensure high quality of cell detections, we applied a two-step verification process of detected objects. Because of the formalin-based fixation, FFPE tissues are known to be highly autofluorescent^26^ along with structural elements such as collagen and elastin increasing such confounding phenomena. Additionally, highly vascularized tissues contain a significant number of erythrocytes that can be encountered in all acquisition channels of COMET microscope, including faint signal in DAPI channel. To discriminate between the true cell detections and artefacts caused by autofluorescent signal, we applied a two-step single-cell data cleaning procedure^27^. Once all measurements were exported, we performed unsupervised clustering for all nucleus features based on the measurements in all channels but DAPI. Using results visualized with UMAP approach (Figure S1A), we could detect 4 clusters that were characterized by high expression of all the markers as well as relatively strong signal in unstained images due to tissue autofluorescence (Figure S1B). Manual curation of these clusters revealed the high levels of erythrocytes’ detection (Figure S1C,D) at each cycle of the seqIF workflow within them and were therefore excluded from subsequent analysis. In the second step of filtering, we excluded objects based on 4 features, (1) model specific feature—StarDist detection probability (cut-off value: 0.65, Figure S2A), (2) signal-based feature—DAPI mean intensity (cut-off value: 2917.1, Figure S2B), and shape-based features— (3) nucleus area (7.1 µm^2^ < accepted value < 137.9 µm^2^, Figure S2C) and (4) nucleus circularity (cut-off value: 0.65, Figure S2D). Visual inspection confirmed that excluded objects were mostly artefacts (Figure S2E, F). In total, 14,229 cells were discarded during the 2 steps of data cleaning with 55,063 cells passing the quality control and deemed as acceptable for the subsequent analysis pipeline.

Before the final step of feature extraction, background subtraction was performed for all channels separately, using the corresponding autofluorescence channel recorded before each cycle^9^. The background subtraction was performed pixel-wise and infrequent negative pixel values were zero-floored.

### Dynamic range assessment

To investigate in detail the biomarker expression within the tissues, a single-cell resolution of the image as well as a broad dynamic range of the immunofluorescent signal must be delivered by imaging modality. COMET images have a pixel size of 0.23 μm and a spatial resolution below 1 µm, which is sufficient to clearly discriminate the subcellular biomarker expression patterns and segment cells into their nuclear, cytoplasmic, and membranous compartments (Fig. 2E,F). To investigate if images generated with COMET provide a dynamic range of fluorescent signal sufficient to discriminate different levels of biomarker expression, we examined in more detail the HLA-DR expressing cells in the lung tumor metastasis core of specimen 3 (core C2, Figure S3). HLA-DR protein expression is known to be reflecting the activation status on immune cells as macrophages and dendritic cells^28^. HLA-DR expression can also be triggered in the tumor cells^29^, thus its expression levels are expected to be heterogeneous and can vary from negative through low, medium, and high.

In this specimen, we identified a bimodal expression of HLA-DR with high levels expressed by immune cells and low expression found in epithelial tumor cells (Figure S3A). When exploiting the mean cell intensity as a unique parameter to interrogate cell phenotypes, we found distinguishable differences between the two cell types (Figure S3B). When mean cell intensity was compared with the cell size feature on the biaxial scatter plot, both the identification and quantification of immune vs tumor cells could be straightforwardly achieved with a gating strategy (Figure S3C). These data demonstrate that COMET platform has a sufficient resolution and dynamic range to accurately discriminate cell-intrinsic biomarker expression variability.

### Supervised phenotyping of tumor microenvironment

**Figure 3:**
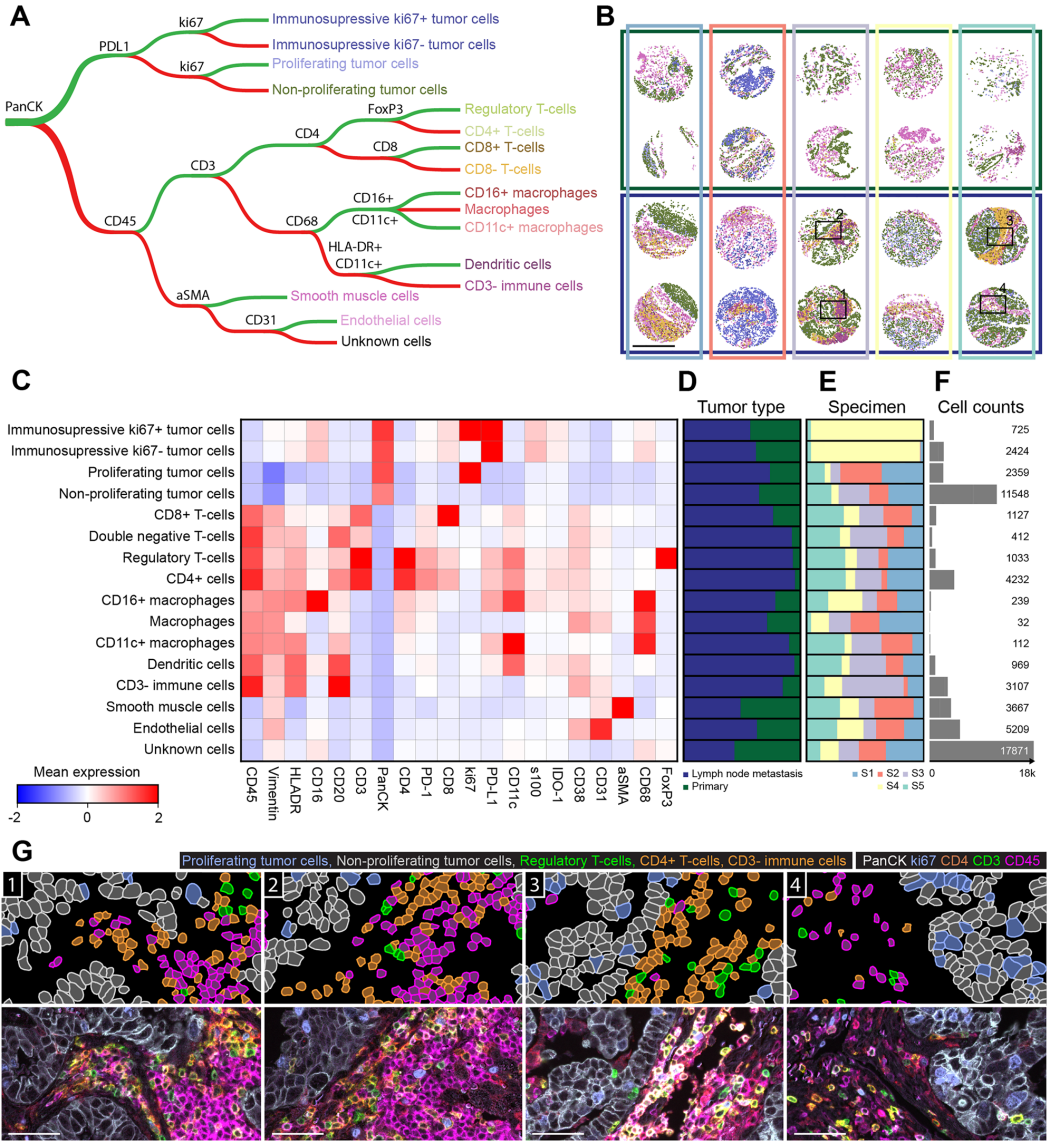
Supervised rule-based cell phenotyping assisted with automatic thresholding can be applied to the COMET dataset to identify known phenotypes present in the data and to quantify their populations. (**A**) A rule-based tree classifier was used to assign cell types to individual cells based on their binary expression profiles. The expression was binarized (i.e., positive vs negative label for each marker) using an automatic thresholding approach described in Figure S4. (**B**) Spatial distribution of all cell types found using the tree classification shown in (**A**). The cells classified as unknown are not visualized. The colors of horizontal rectangles show the different tumor types (primary versus lymph node metastasis) as in (**D**) and vertical rectangles refer to different specimens (sample S1–S5) as in (**E**). The rectangles marked with numbers 1–4 correspond to the zoom-ins in panel (**G**). The scale bar: 500 µm. (**C**) Expression matrix for each of the cell phenotypes presented in (**A**). Z-normalized mean expression values per group are visualized. (**D**,**E**) Normalized cell type distributions of corresponding cell phenotypes for (**D**) different tumor types: primary vs metastatic and (**E**) for different specimens: S1–S5. (**F**) Cell count histogram for corresponding cell phenotypes. (**G**) Annotations of classified tumor and immune cells (top) and the corresponding antigens detected in the multi-color fluorescence images (bottom). Scale bars: 50 µm.

Spatial detection of multiple biomarkers enables the identification of diverse cell types present within a tissue. To spatially find predefined cell types based on known expression patterns^30^, we applied supervised methods based on a priori classification rules as a first approach (Fig. 3A). The 20-plex panel presented here was established with the aim to characterize tumor-infiltrating immune cells within the TME (Fig. 2C). The panel was designed to allow performing a rule-based single-cell phenotyping which uses binary expression features derived with a threshold-based approach (Fig. 3A). We characterized the immune cell infiltration level, along with tumor-intrinsic features and stromal compartments of different cores of a lung cancer TMA.

In the first step of data analysis, we z-normalized mean cell intensities for all 20 biomarkers (Figure S4A,B). Subsequently, we applied an automatic threshold-based approach to determine positive cells for each marker individually. We established a metrics-based approach relying on the statistical characteristics of the background signal (i.e., negative cells) (see Methods chapter: Supervised phenotyping and Figure S4C for more details). This semi-automatic thresholding approach was deployed to eliminate the user bias and harmonize thresholding values over all markers. Our approach successfully detected the positive cells (Figure S4D,E), which was further confirmed by the visual inspection by experienced senior biologists. To identify distinct cell types, we applied a decision tree-based classification (Fig. 3A). Cell identities were manually assigned based on the known marker combinations, which are established in literature^31^. Based on this approach, different cellular classes were detected in most of the cores present in the TMA (Fig. 3B–F). Unknown cells, that did not fit any of the identified classes, were summing up to 30% of all cells and were mainly present in primary tumor cores (Fig. 3B,D) that displayed lower expression levels for markers of interest.

The second most abundant class was a cell type with a predefined phenotype of PanCK+ non-proliferating tumor cells. Visual inspection of randomly selected areas of the TMA confirmed that once the cell type was identified, the phenotyping of the cells was accurate, and it properly reflected the biomarker signal (Fig. 3G). The main limitation of a rule-based classifier stems from the lack of inclusion for the markers lying outside of the established rules. To examine the expression patterns of each pre-defined class, we plotted a heatmap showing the biomarker abundance and distribution for the detected cell types (Fig. 3C). We could confirm the expected expression of Vimentin positivity by immune and stromal cells and its absence within tumor cells. Surprisingly, other markers like CD20, were not limited to CD3- immune cells but also detected to a lower extent in other immune subtypes such as several myeloid and T cell classes. Indeed, for small densely clustered cell types such as lymphocytes, signal spillover through cell masks is one of the most important challenges in threshold-based classification^32^. Indeed, to minimize the signal leak from neighboring cells into an area used for the phenotyping of T cells, we analyzed the mean intensity of CD3, CD4, and CD8 markers within the nucleus mask and a similar approach for other small cells, as B cells, might help in their threshold-based phenotyping.

Additionally, we have also tested the recently published Astir algorithm as an alternative to developing a fully automated threshold-based pipeline for cell identification^33^. This machine learning algorithm was developed to provide unbiased classification of cells into predefined classes^33^ and can be easily applied to COMET image-derived data. We aimed to detect the same classes as in our decision tree-based classifier (Figure S5A). Astir algorithm detected unknown cells with the highest frequency, especially in primary tumor cores, while immunosuppressive tumor cells were the second most frequent cell type identified (Figure S5B–D). Similarly, to the threshold-based classifier, the simple phenotypes were assigned as expected with a few rules (Figure S5A), while the assignment of complex phenotypes, (i.e., dendritic cells) turned out to be more challenging.

### Unsupervised cell classification and spatial cell distribution

**Figure 4:**
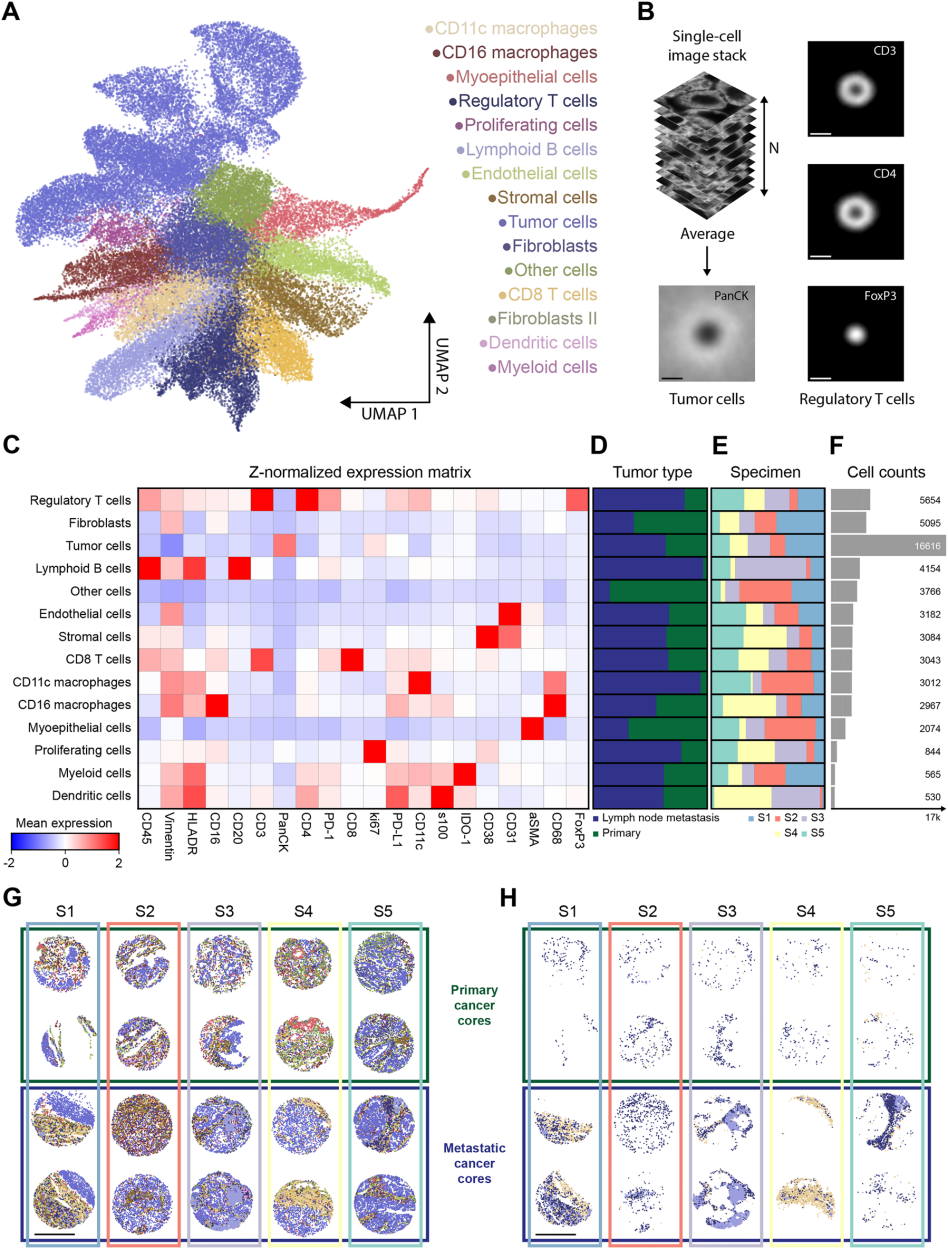
Unsupervised cell phenotyping using Leiden clustering and assisted by UMAP performs fast and unbiased mapping of the single-cell phenotypes present in the COMET dataset and enables subsequent quantification of tissue composition. (**A**) UMAP projection of all markers with reference cell phenotypes assigned based on the Leiden clustering results. Further details on cell phenotype assignment can be found in  Methods chapter: Unsupervised phenotyping and SI Figs. 7–9. (**B**) Quantification of single-cell expression patterns at sub-cellular scale. Averaging of single-cell image crops was performed to obtain average expression profiles for the randomly selected subset (N = 2000) of tumor cells (PanCK) and regulatory T-cells (CD3, CD4, FoxP3) selected with an identical approach. Scale bars: 5 µm. **C**) The expression matrix for each of the cell phenotypes based on Leiden clustering results. Z-normalized mean expression values per class are visualized. (**D**,**E**) Normalized cell type distributions of corresponding cell phenotypes for (**D**) different tumor types (primary or metastatic) and (**E**) for different specimens. (**F**) Cell count histogram for corresponding cell phenotypes. (**G**) A spatial distribution of all cell phenotypes with colors corresponding to Leiden classes. (**H**) A spatial distribution of regulatory T cells, lymphoid B cells and CD11c macrophages, showing the apparent density difference between primary and metastatic tumors. Scale bars in (**G**,**H**): 500 µm.

After evaluating how supervised classification methods can be applied to a COMET dataset, we explored unsupervised classification as an alternative automated workflow (Fig. 4). We performed Leiden clustering^34^ and UMAP dimensionality reduction technique^35^ for data visualization.

Leiden clustering resulted in the detection of 20 clusters (Figure S8), that were merged into 14 clusters (Fig. 4A, see below) after detailed examination and analysis as described below. For each cluster, we further examined the expression patterns of the corresponding signature markers—for example, for the regulatory T cells cluster we generated a mean intensity projection for the signature markers of cells belonging to this cluster, where the expected localization of FoxP3, CD3 and CD4 expression was confirmed (Fig. 4B). Similarly, we could identify the exclusive cytoplasmic expression of PanCK with no nuclear interference (Fig. 4B). It further demonstrates that the spatial resolution obtained is sufficient to quantify the spatial biomarker expression at a sub-cellular level. Additionally, the known expression patterns can be used for optimization and quality control of unsupervised cell approximation algorithms.

Following the unsupervised classification step, cell identity for each class was assessed based on the following parameters: (1) expression level of the markers in corresponding sub-cellular compartments (Table 1) in each of the clusters (Fig. 4A,C, Figure S7), (2) visual inspection of the cells in the tissue context, and (3) literature reference. Using this method, 6 clusters expressing heterogeneous levels of PanCK were identified, all of them located nearby in the UMAP representation graph (Figure S8A,B). Therefore, we merged these clusters into a metacluster of tumor cells (Figure S8C). As a result, the tumor cell cluster was the most abundant in this dataset, which is expected, considering that 39% of the original image area is PanCK positive (PanCK+) (See details in the Methods chapter: Image segmentation). However, the total stromal components outnumbered the number of the PanCK+ cells corroborating the high infiltration of non-tumoral cells previously reported for lung tumors^36,37^. Some clusters were consistently detected in all specimens, with a higher frequency of activated T cells, B cells and the CD11c+ macrophages being present in the secondary tumors. Importantly, the spatial evaluation of the clusters revealed the degree of tissue heterogeneity and the different patterns of immune infiltrations between specimens of primary and metastatic cancer tissues (Fig. 4G,H).

**Figure 5:**
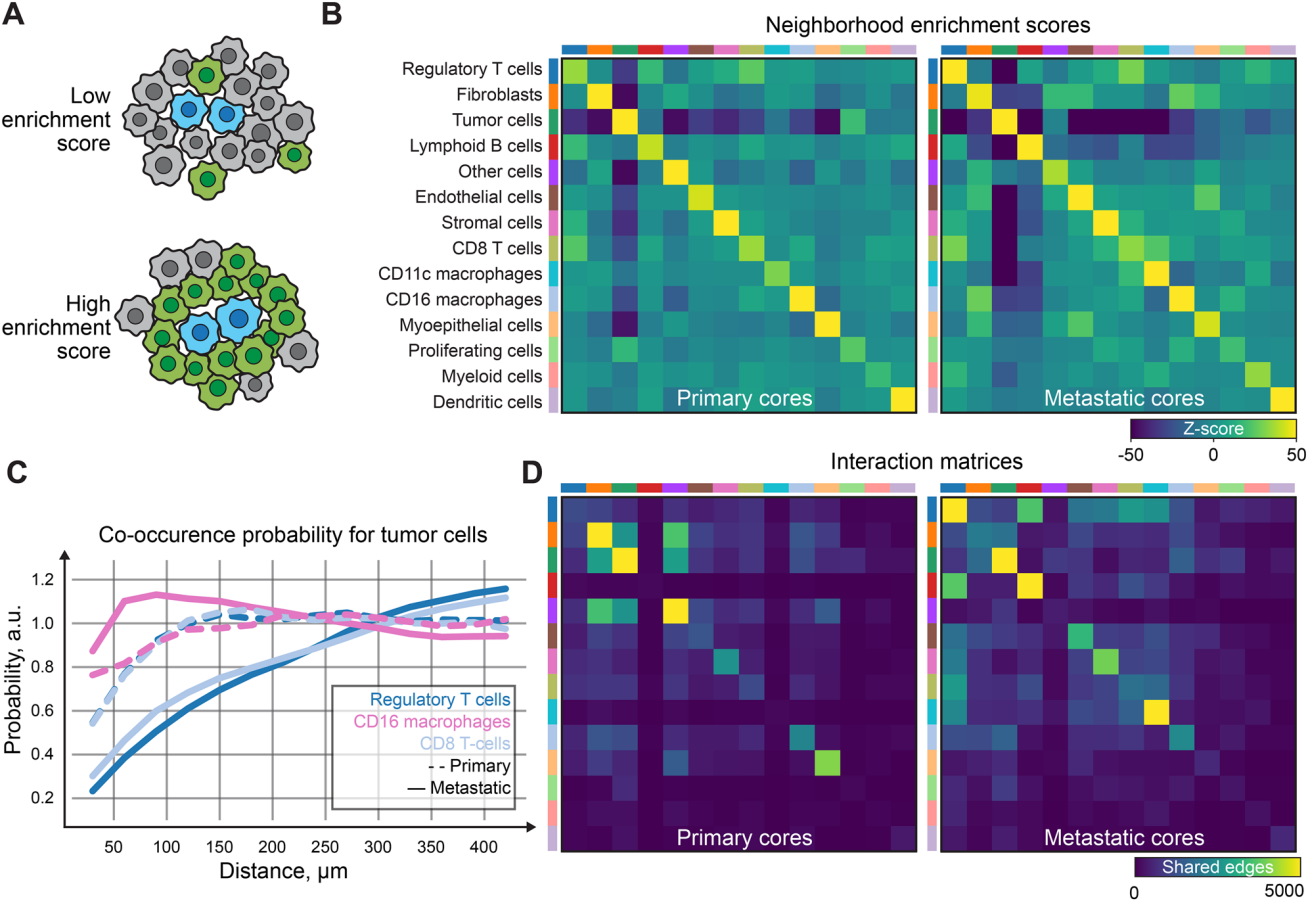
Spatial analysis of the tumor microenvironment reveals spatial connections between interacting cell phenotypes identified with the unsupervised clustering results. (**A**) A schematic explanation of the cellular neighborhood enrichment score which was used to establish and quantify spatial relations between different cell phenotypes. Enrichment score is a metric that quantifies the degree to which cells from one cluster are frequently close to cells from another cluster. A high score indicates enrichment, while a low score indicates depletion. Blue: cells of interest, green: cell class being analyzed as a neighboring class, gray cells: other cells surrounding cells of interest. (**B**) Neighborhood enrichment scores for different cell phenotypes for primary (left) and metastatic (right) tumor tissues. (**C**) Co-occurrence probability between tumor cells and regulatory CD4+ T cells, CD16+ macrophages and CD8+ T cells plotted vs the distance from the tumor cells. (**D**) Interaction matrices, showing the number of shared edges in between cells from different phenotypes. Matrices for phenotypes from primary (left) and metastatic (right) tumor tissues are displayed.

Once the phenotypic classes were properly assigned to each cluster identified in Fig. 4, we investigated the degree of cell proximity and interaction to identify interacting cells in the analyzed COMET dataset. Due to a large fraction of unknown cells in the supervised approach, we have decided to perform the spatial examination of the clusters identified via the unsupervised clustering approach. We applied spatial characteristics such as cellular neighborhood enrichment score and the co-occurrence probability (Fig. 5A–C). Due to the small size of the TMA cores, the results are not fully representative of the original tissue milieu, however, we could observe, that macrophages tended to localize to a greater extent in proximity to the tumor cells (Fig. 5C), while T cells tended to remain further from tumor cells. Interestingly, the distribution of T regulatory cells seemed to differ between primary and metastatic tumors with more frequent homotypic T regulatory cells’ neighborhood (Fig. 5B) and intracellular interactions (Fig. 5D) in metastatic cores. Tumor cell homotypic interactions, reported previously^38^, were also detected in our dataset (Fig. 5B,D). Preliminary observations on spatial characteristics highlighted the potential of a spatial analysis approach to identify tissue-specific patterns of cell distributions.

## Discussion

In the past decade, spatial biology emerged as an important tool in understanding tissue biology^39^. Several novel approaches to interrogate the protein composition of tissue have recently been developed^40^ including, but not limited to: cyclic immunofluorescence^41^, co-detection by indexing^42^, multiplex ion beam imaging^43^, seqIF^9^, in addition to manual immunofluorescence protocols as for example iterative indirect immunofluorescence imaging^44^. Automation, together with protocol improvements, significantly decreased the turnaround time of staining and imaging experiments, as demonstrated by the COMET platform, presented here, enabling large cohorts to be processed with multiple biomarkers in weeks instead of months. Alongside high acquisition speed, COMET platform can acquire data with sub-µm resolution and sub-pixel image registration accuracy, thus ensuring high-image quality for downstream data analysis. This unseen pace of data acquisition imposes an important need to establish intuitive, fast, and easy-to-adapt image analysis pipelines to process, analyze and interpret the data. Assay automation tends to decrease the risk of technical variability in the data generation pipeline^9^. However, the risk of user-induced bias in the image analysis should also be considered as an important parameter when evaluating and comparing spatial biology results^45^, especially when applied to clinical outcomes such as predictive responses or prognosis. The hyperplex datasets are exceptionally prolific in information about tissue biology but data interpretation still requires investments in terms of resources, infrastructure, and time, especially when applying in-depth image analysis methods. Consequently, image processing often becomes a bottleneck, negatively impacting the efficiency of research studies. Thus, active implementation of fully automated and unbiased workflows is on the surge and pushing forward the boundaries of digital tissue pathology^7,21^. In the study presented here, we show compelling evidence that seqIF on COMET enables a detailed tumor microenvironment analysis and provides fertile ground to investigate in depth any tissue composition at single-cell level and with subcellular resolution. To allow the spatial biology field to overcome the persistent challenges for data analysis and interpretation, the success of protocol automation needs to be transferred to image analysis.

To facilitate the automation of image analysis on complex immunofluorescence datasets, certain preprocessing and data filtering steps might be performed upfront. The common practice is to decrease the contribution of autofluorescence in the analyzed signal, either during the sample preparation and/or at the data acquisition step^46^. Additionally, during the image analysis, the background signal can be removed digitally once the assay is completed by applying a pixel-wise background subtraction step with a corresponding autofluorescence image of the same tissue. COMET enables the simultaneous application of all these methods of background subtraction aimed to significantly increase the signal-to-background ratio of immunofluorescence tissue images^9^.

Here, we exploited a priori knowledge of tissue autofluorescence to generate an innovative approach for detecting and filtering out common artifacts at the single-cell level such as the ones caused by erythrocytes, which usually confound the interpretation of tissue geometry when applying nuclei segmentation algorithms. We took advantage of Leiden clustering to successfully find false positive single cell candidates that were then visually identified as erythrocytes. Such automated procedure does not require laborious pre-training as being completely data driven. Thus, it allows to efficiently filter out false positive detections that can be mistakenly interpreted as rare cell subtypes at a later stage of the analysis. Thereafter, we also performed common data cleaning operations to reduce the number of false positive detections. Our approach proved the advantage of data-driven pre-processing and highlighted it as being a crucial step to minimizing the error rate for the downstream single-cell analysis^40,47^.

Determining cell identities with the use of known expression patterns is currently the gold standard method for cell identification in the field of single-cell analysis^48,49^. Such rule-based supervised approach is largely used in flow cytometry^50^ and in time-of-flight mass spectrometry^51^, and has been further implemented by the digital pathology field. For example, to identify PD-L1 positive cells as “companion” or “complementary” diagnostics, tumor proportional score or tumor cell expression is being leveraged^52^. While supervised classification is very efficient for low-plex images and for a small number of rules, its deployment for hyperplex data remains cumbersome, due to the growing complexity of analysis and to the labor-intensive set up of the rules. This consideration is further corroborated by the need for manual or semi-automatic thresholding, which becomes challenging for heterogeneous datasets entailing multiple tissue samples, such as TMAs. Furthermore, manual adjustments of marker positivity thresholds often result in user-specific bias. To mitigate the above-mentioned issues, we applied an automatic thresholding approach for rule-based classification. The FFeX technology provides uniform staining within the whole staining area, ensuring that the detected fluorescence signal reflects biomarker expression^25^. However, due to the inter-sample variability in our dataset, we could detect a wide range of biomarker signal intensity, and therefore, a large fraction of cells was not classified. Interestingly, we observed that the supervised phenotyping outcomes produced by Astir and the threshold-based classifier were both strongly dependent on the rule complexity, and therefore, class distribution was unbalanced quantitatively. Thus, the thresholding and classification might need to be separately adapted per specimen for adapting our approach to tissue-intrinsic variability of biomarker expression. Overall, the general rule-based classifier which uses global thresholding might not be fitted for analyses of a heterogenous set of samples even when automation decreases the assay variability. The versatility offered by automating technologies has surged the portfolio of biomarkers spatially detected, further highlighting the need for establishing classification guidelines aimed at harmonizing single-cell spatial analytics^4,40,47^.

Unsupervised cell phenotyping mitigates user-based bias and enables full immersion in the data without a priori assumptions, and mostly relies on the choice of clustering method parameters, the input data filtering in addition to the data normalization approach used^47,53^. The development of clustering methods applied to high-parameter single-cell data analysis is evolving at a frantic pace, and the more recent approaches aim to minimize the number of prior assumptions^54^ while exploiting the overall data structure^55^ and/or the statistical cluster robustness^56^. Interestingly, it was shown that the data normalization process of hyperplex datasets significantly confounds the outcomes of classic unsupervised clustering^53^, spotlighting that the more appropriate normalization method should be accurately identified.

In addition, we observed that heterogeneous datasets bear remarkable levels of intra-sample variation at the biomarker expression level (Figure S8) as well as intra-population variation between detected cells (Figures S3, S8) thus prompting the appearance of biologically irrelevant clusters that required a meta-cluster assignment process via manual curation (Figure S8). These overarching issues can possibly be mitigated with the use of novel data-driven sample normalization methods^57,58^. As an exploratory single-cell data analysis approach, unsupervised clustering still requires accurate data preparation, cross-validation of the outcomes and often the manual visual inspection of cluster identity. Once the automation of cluster translation into cell types is ready, the full potential of unsupervised cell phenotyping could be unlocked and free studies from user-driven interpretation biases.

The extraction of biological signatures and/or clinical scores from hyperplex tissue images generates a new dimension for the spatial profiling of single cells. However, this mining process can only be achieved once the cell types are correctly identified, because spatial metrics are typically computed as a final step of the analysis. Here, the proposed open-source tools are easily applicable to data obtained from automated hyperplex platforms such as COMET. We deployed cellular neighborhood and interaction analysis approaches (Fig. 5) to reveal a detailed cellular mapping of tissue specimens^59^. Our analysis corroborated on previous observations^38^ that lung tumor cells prioritize homotypic interactions, despite using a very heterogeneous dataset limited in size.

The field of spatial biology is rapidly expanding from research-use-only applications into a more clinically relevant angle where spatial biomarkers could represent a novel diagnostic toolkit in both prognostic and predictive settings. Recently, AI-driven image analysis applied to spatial cellular contexts has shown a possibility of accurately predicting lung tumor clinical outcomes from biopsies, thus demonstrating a strong potential for clinical use^38,60^. In addition, spatial localization metrics such as PhenoTIL, which combines hematoxylin and eosin staining with multiplex immunofluorescence images, has been shown as a novel predictor score for lung cancer^61^. Along with other recently developed analogies, this successful approach showcased its potential to illuminate unappreciated biological mechanisms that can lead to more accurate diagnostic biomarkers.

New imaging technologies allow for unprecedented access to information detailing a myriad of biological processes thus generating cellular and tissue atlases related to research and therapeutic discoveries. To accomplish the promises of spatial biology, technical challenges related to data acquisition, processing and interpretation must be addressed and the handling of such complexity will strongly depend upon the integration of computational approaches seamlessly operating across multi-layered datasets. On top of that, we believe that the scientific community shall continue to profuse efforts towards multi-site cross-validation of fully automated image analysis workflows ideally across different immunofluorescence-based modalities. The need for democratizing, validating, and scaling up image analysis workflows is pressing, and this advancement will help to elevate spatial biology into a technology that is poised to become harmonized and capillary, revolutionizing the research and clinical domains.

### Supplementary Information

Supplementary Information.

## Data Availability

Background-subtracted raw TMA OME-TIFF image is available under the link: https://lunaphore.com/download-center-tma-downstream-analysis/. Scripts and single-cell data are available in the GitHub depository under the link: https://github.com/lunaphore-public/downstream-analysis-toolbox.
