# Supplementary material for: Semi-automated approaches for interrogating spatial heterogeneity of tissue samples
Source: Sci Rep. 2024 Feb 29;14:5025. doi: 10.1038/s41598-024-55387-w (PMC10904364; doi:10.1038/s41598-024-55387-w)
Supplement: Supplementary file 1 — Supplementary Information. [file 41598_2024_55387_MOESM1_ESM.pdf]

# Table of Contents


Title ........ 2

Author information ........ 2

Affiliations ........ 2

Corresponding author ........ 2

Supplementary Figures and Legends ........ 3

Supplementary Figure 1 ........ 3

Supplementary Figure 2 ........ 4

Supplementary Figure 3 ........ 5

Supplementary Figure 4 ........ 6

Supplementary Figure 5 ........ 8

Supplementary Figure 6 ........ 9

Supplementary Figure 7 ........ 10

Supplementary Figure 8 ........ 12

Supplementary Figure 9 ........ 13

References ........ 14

## Title

Semi-automated approaches for interrogating spatial heterogeneity of tissue samples

## Author information

Vytautas Navikas, Joanna Kowal, Daniel Rodriguez, François Rivest, Saska Brajkovic, Marco Cassano, Diego Dupouy

## Affiliations

Lunaphore Technologies SA, Tolochenaz, Switzerland

## Corresponding author

Correspondence to diego.dupouy@bio-techne.com

# Supplementary Figures and Legends

## Supplementary Figure 1

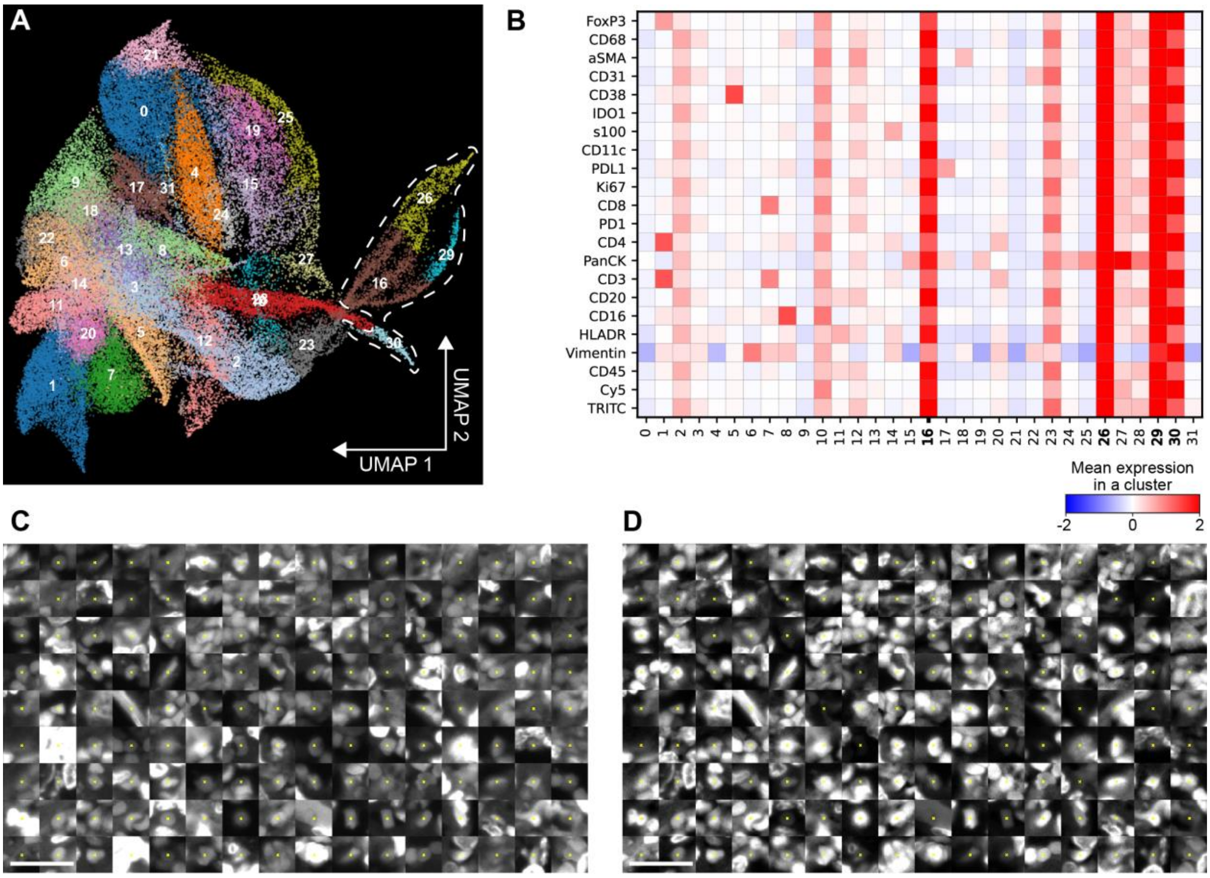


**Supplementary Figure 1.** Erythrocytes can be detected and excluded from the analysis of the COMET dataset.

**(A)** UMAP of single-cell mean intensities extracted from the WSI image stack with no background subtraction applied. Clusters found by the Leiden clustering algorithm are shown in different colors. The Leiden clusters that are associated with erythrocytes are marked with a dashed line.

**(B)** We consider that the detected cells, that were expressing all the markers and had a high-intensity fluorescence signal in both autofluorescence channels (Cy5 and TRITC) should be excluded from further analysis. Based on morphology, the cells were identified as erythrocytes. They were present in all TMA cores.

**(C-D)** Non-normalized (C) and crop-wise normalized (D) image crops for erythrocyte candidates that were excluded from the analysis. In total, 3,432 cells were excluded from the further downstream analysis by the method described. Scale bars in C and D are 20 µm.

## Supplementary Figure 2

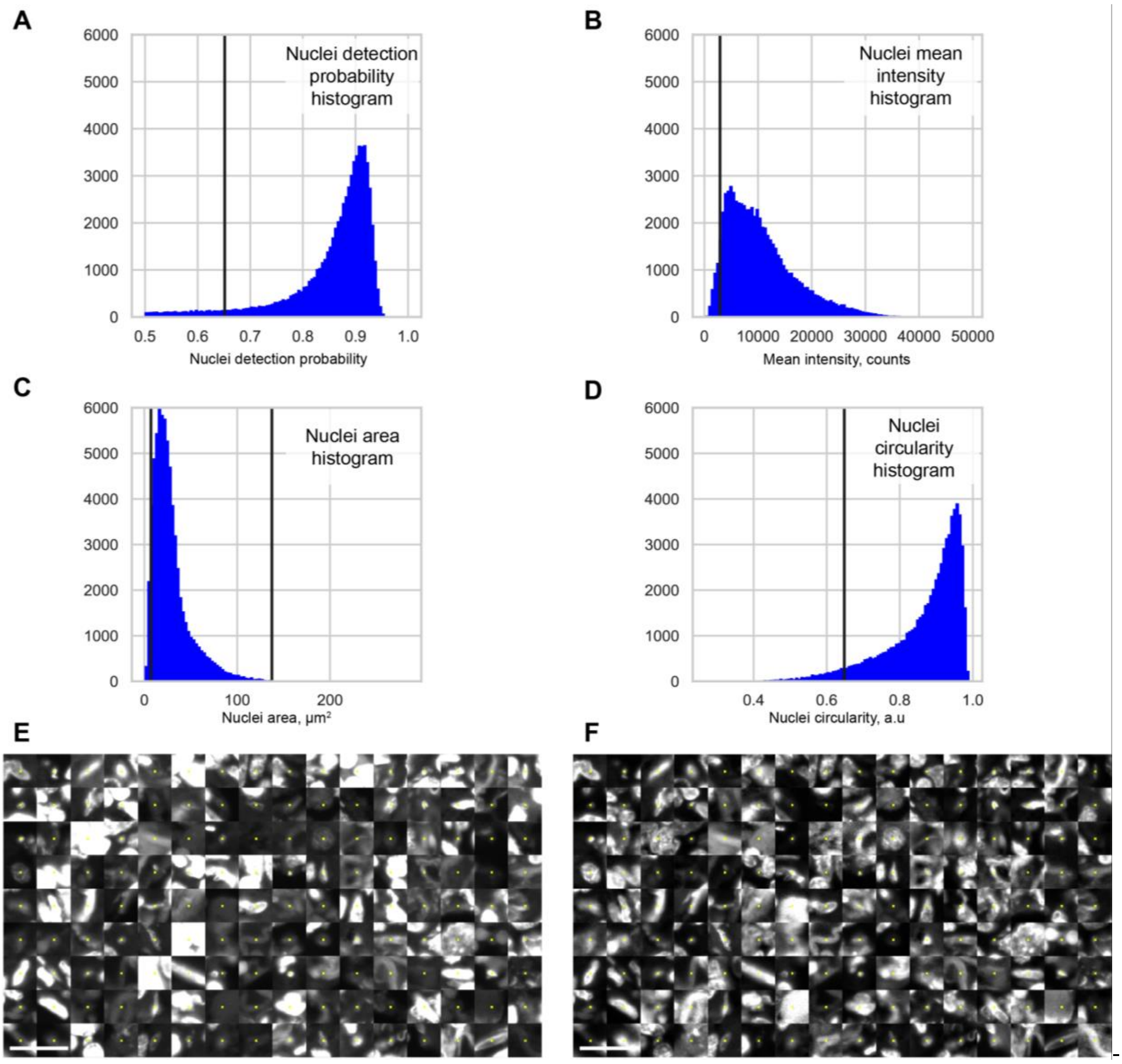


**Supplementary Figure 2.** False positive nuclei segmentations can be excluded from the analysis based on DAPI and area features.

**(A-D)** Histograms of different features used for post-filtering of detected nuclei. Histograms were gated based on the 0.05 and 0.999 (only area) quantiles. Gating ranges are marked with black lines.

**(E-F)** Non-normalized (E) and crop-wise normalized (F) image crops representing the excluded nuclei. In total, 10,797 cells were excluded from further analysis by the described procedure. Scale bars in E and F are 20 μm.

## Supplementary Figure 3

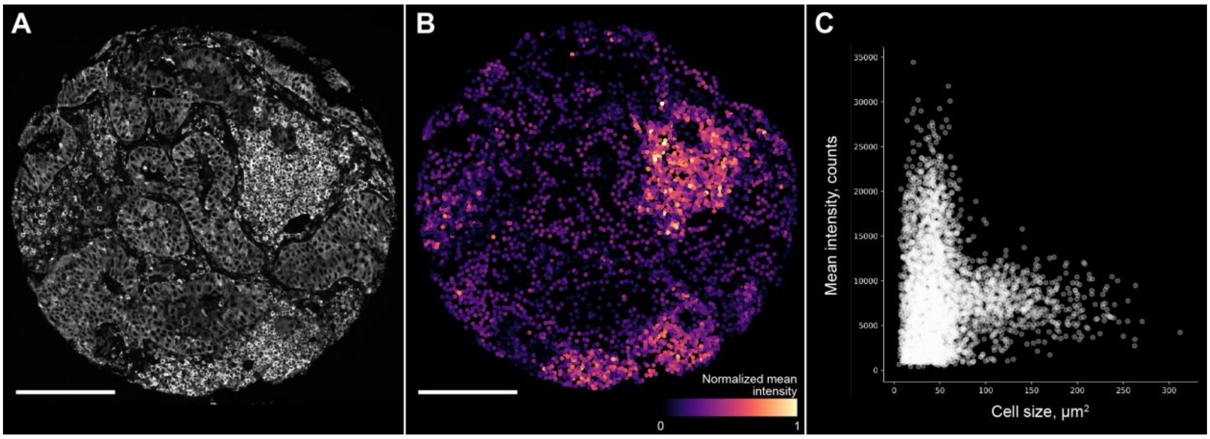


**Supplementary Figure 3**. A wide dynamic range of signal intensity allows to differentiate cell populations.

**(A)** A single-channel image of an HLA-DR marker. The scale bar is 200 μm.

**(B)** The same image normalized from 0 to 0.99 for visualization purposes only. The scale bar is 200 μm.

**(C)** A scatter plot displaying mean cell signal intensity from the corresponding image (A) vs cell size estimated as the area of a dilated nucleus as described in the Methods. Scatter plot visualization allows for easy discrimination of two subpopulations of HLA-DR$^+$ cells: highly expressing small cells and low expressing large cells, highlighting the potential of COMET derived dataset to discriminate different levels of expression of biomarker when performing cell-based analysis.

## Supplementary Figure 4

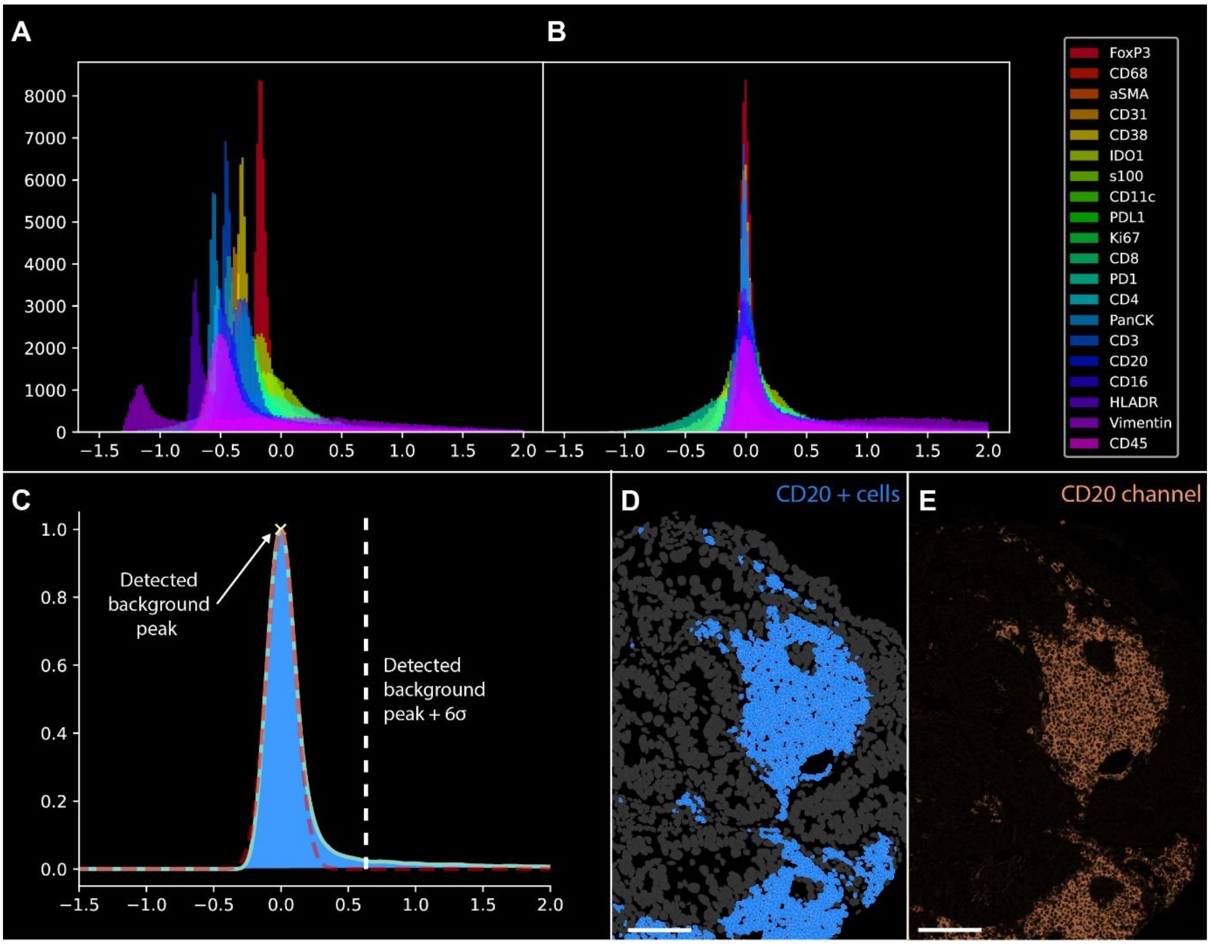


**Supplementary Figure 4.** Automatic thresholding of single-cell expression data based on the background signal allows to automate cell identification.

**(A)** Z-normalized distributions of mean cell intensities of all 20 markers from the hyperplex panel.

**(B)** To perform automatic thresholding, the signal was divided into negative and positive signals. Background intensity values were inferred as a negative signal. The background was detected as the left peak in a Z-normalized histogram of mean cell intensities. Single-cell mean intensity distributions from all channels are aligned to a background peak for visualization purposes only.

**(C)** Representative single-cell mean intensity distribution (CD20) centered at the mean background intensity. The threshold was set to +6σ and it is marked with a white-dashed line. The σ was defined as FWHM/2.355 and was calculated for each marker separately. The fitted Gaussian distribution is marked in red, showing a close agreement to a background signal distribution. Since the actual background intensity distributions deviate from a perfect Gaussian distribution, the selected threshold was chosen rather high.

**(D-E)** Positive and negative CD20 cells (D) with a corresponding fluorescence image (E). Scale bars in both images are 100 μm.

## Supplementary Figure 5

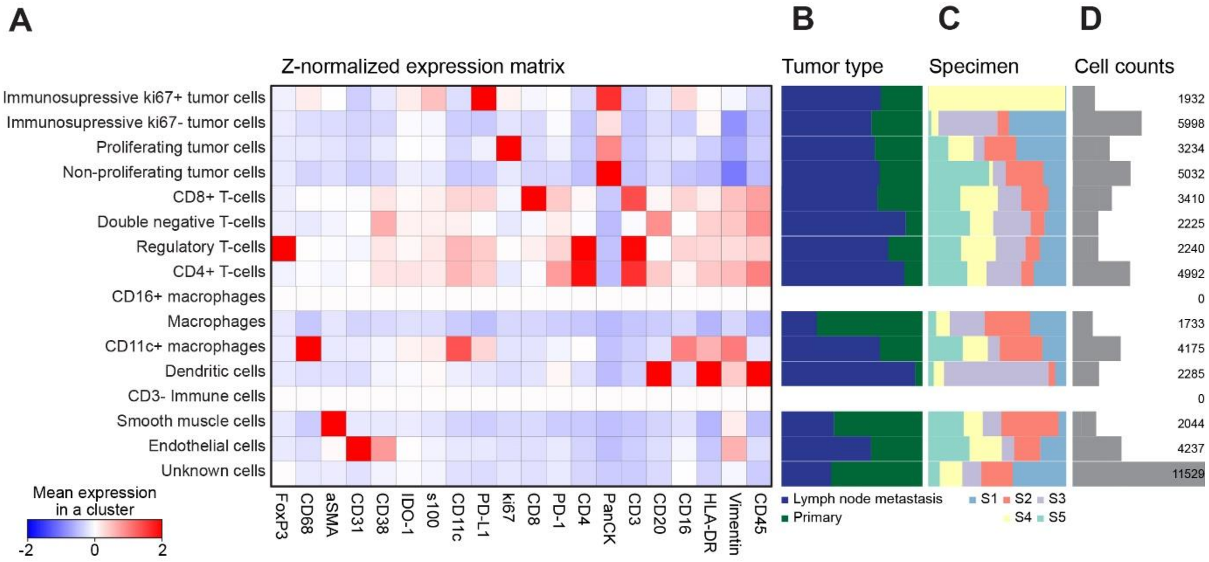


**Supplementary Figure 5.** Automatic cell type assignment using the Astir framework identifies cell subsets in the dataset [1]

For a comparative analysis, the cell types specified in Figure 3A and used for the decision tree-based classifier were also applied for an automatic cell-type assignment with a recently published Astir framework. According to the authors' instructions, instead of z-normalized expression data, we used a global arcsinh normalization with a division factor of 150 for this part of the analysis. The difference in the data normalization method is likely affecting the cell-type assignment results, thus resulting in quantitative differences.

**(A)** A cell-expression matrix of cell-type assignments. Z-normalized mean intensity values are displayed. The cell phenotypes that were not found (N=5) are shown in white.

**(B-C)** Normalized cell type distributions of corresponding cell phenotypes for different tumor types (primary or metastatic) and different specimens.

**(D)** Cell count histogram for each of the phenotypes.

## Supplementary Figure 6

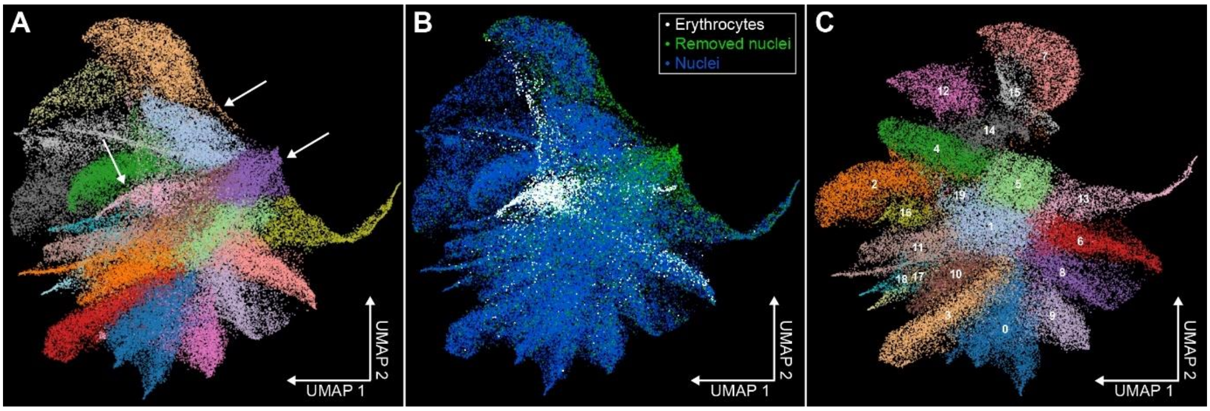


**Supplementary Figure 6.** The proposed data filtering procedure improves Leiden clustering results and reduces the probability of spurious phenotypes.

**(A)** UMAP of all the data, with no filtering applied, containing 68,801 detected cells. Clusters detected with the Leiden clustering algorithm are displayed in different colors.

**(B)** UMAP of all (N = 68,801) cells with different types of nuclei highlighted. Erythrocytes were detected by the procedure described in Figure S1 (White), nuclei removed using a procedure described in Figure S2 (Green) and nuclei used for further downstream analysis (Blue).

**(C)** UMAP of filtered data (N = 55,063 cells). The final UMAP is equivalent to the UMAP in Figure 4A. The same settings for UMAP and Leiden clustering algorithm were used for (A-C), thus demonstrating the impact of data filtering.

## Supplementary Figure 7

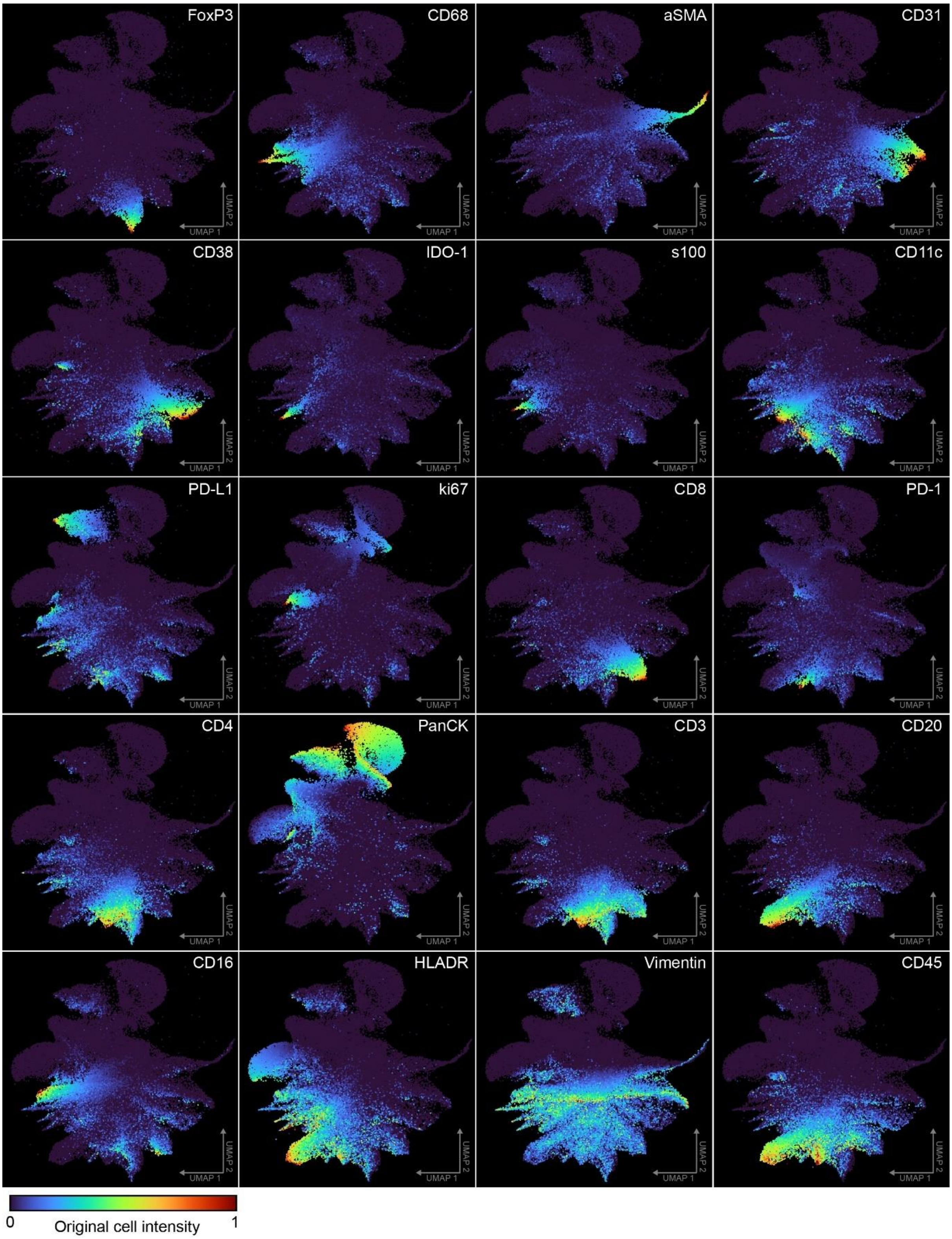


**Supplementary Figure 7.** The expression of each biomarker corresponds to the assigned clusters and confirms their cell phenotype-specific presence.

UMAPs with projected original signal intensities of each channel are displayed. Signals are linearly normalized from 0 to 1.

## Supplementary Figure 8

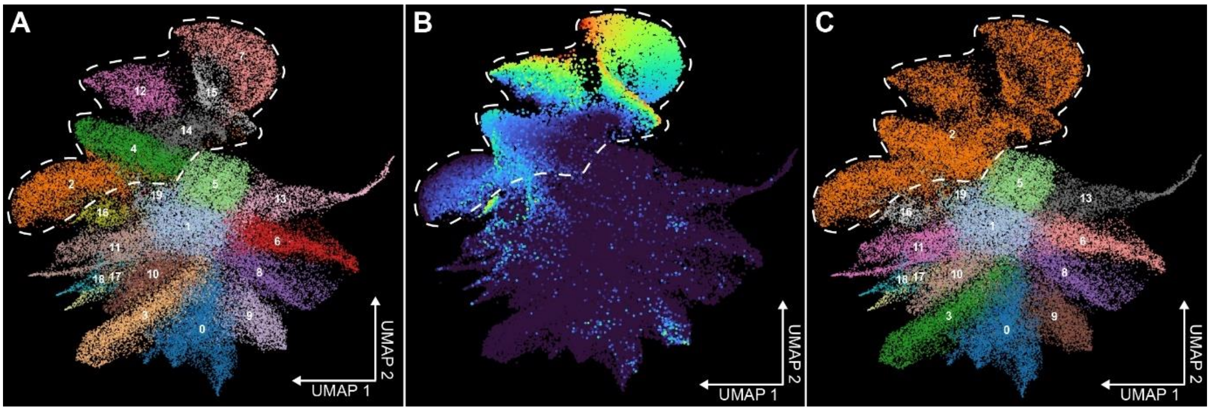


**Supplementary Figure 8.** PanCK-expressing clusters result from inter-specimen variation in biomarker expression and are merged into metacluster based on the expression context.

**(A)** UMAP with original Leiden clusters colored in different colors. The cluster candidates for merging are marked with a dashed line.

**(B)** Original PanCK expression values were linearly normalized from 0 to 1 and projected on UMAP. The scale can be found in SI Figure 7. Clusters 2, 4, 7, 12, 14, 15 have the dominant PanCK expression. It was also found that differences in expression intensity are coming from inter-specimen variation. To minimize inter-specimen variation and its influence on further conclusions, the clusters were merged into one.

**(C)** Final UMAP, which is displayed in Figure 4A with merged PanCK clusters marked with a dashed line.

## Supplementary Figure 9

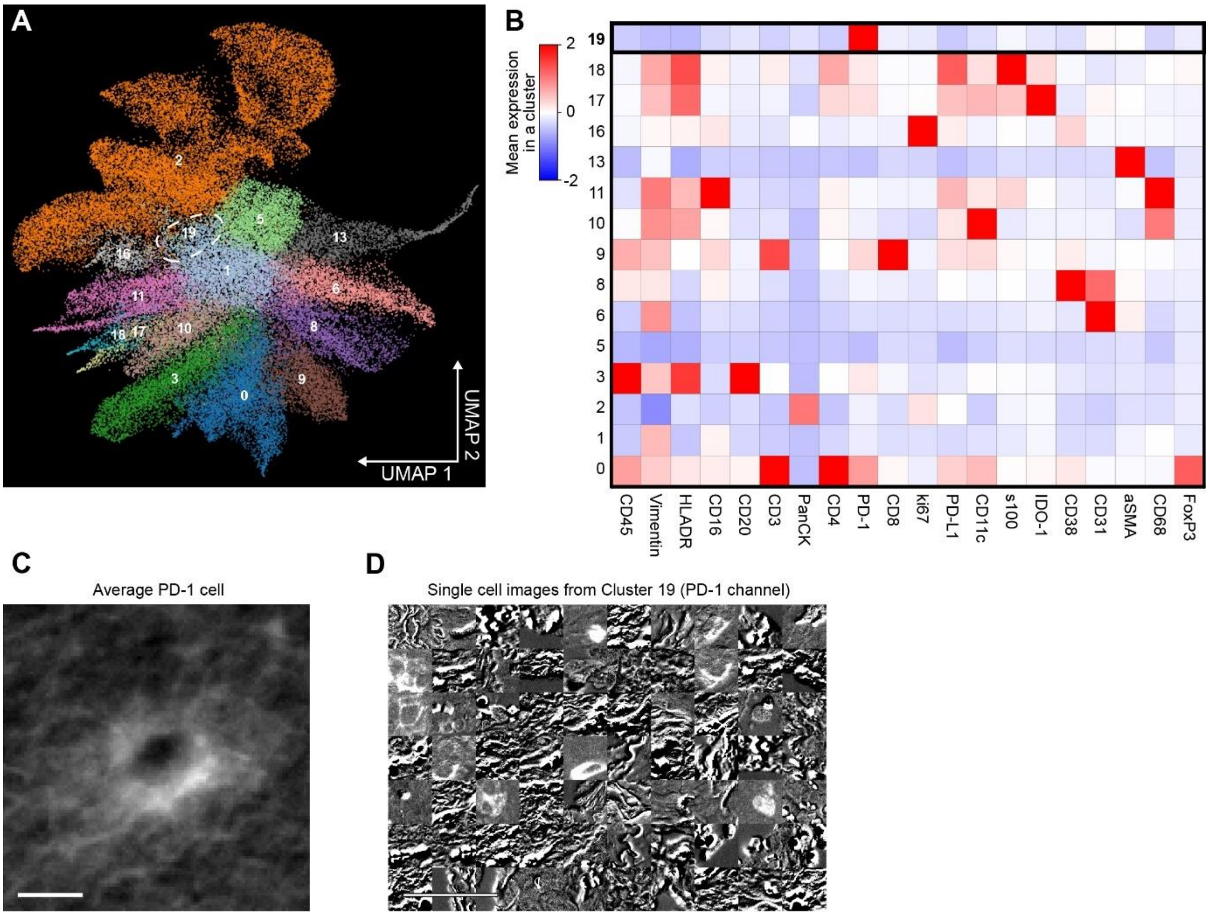


**Supplementary Figure 9.** An in-depth analysis of the expression patterns combined with spatial context allows to identify clusters with false positive marker expressions.

**(A)** UMAP from SI Figure 5C with a cluster of interest marked with a dashed line. The localized cluster of PD-1 expression is further seen in SI Figure 7.

**(B)** Corresponding expression matrix with a cluster of interest (19) marked with a black square. The cluster is dominated by the PD-1 expression. Z-normalized mean intensity values per group are displayed.

**(C)** The averaged cell, using a PD-1 channel image crops (N=1,000). Scale bar is 5 µm.

**(D)** Single-cell crops, with intensities normalized for each image. The morphology of the crops indicates the apparent artifacts that are likely related to background subtraction. The indicated scale bar is 50 µm.

## References

[1] Geuenich, M. J. et al. Automated assignment of cell identity from single-cell multiplexed imaging and proteomic data. *Cell Syst.* **12**(12), https://doi.org/10.1016/j.cels.2021.08.012 (2021).
